# A Novel and Practical Scheme for Resolving the Quality of Samples in Background Modeling

**DOI:** 10.3390/s19061352

**Published:** 2019-03-18

**Authors:** Guian Zhang, Zhiyong Yuan, Qianqian Tong, Qiong Wang

**Affiliations:** 1 Guangdong Provincial Key Laboratory of Computer Vision and Virtual Reality Technology, Shenzhen Institutes of Advanced Technology, Chinese Academy of Sciences, Shenzhen 518055, China; zga@whu.edu.cn; 2 School of Computer Science, Wuhan University, Wuhan 430072, China; qqtwhucs@gmail.com

**Keywords:** bi-variance, convergent frame, denoising, pixel histogram, background modeling

## Abstract

In view of its important application value, background modeling is studied so widely that many techniques have emerged, which mainly concentrate on the selections of the basic model, the granularity of processing, the components in a framework, etc. However, the quality of samples (QoS) for training has long been ignored. There are two aspects regarding this issue, which are how many samples are suitable and which samples are reliable. To tackle the “how many” problem, in this paper, we propose a convergent method, coined Bi-Variance (BV), to decide an appropriate endpoint in the training sequence. In this way, samples in the range from the first frame to the endpoint can be used for model establishment, rather than using all the samples. With respect to the “which” problem, we construct a pixel histogram for each pixel and subtract one from each bin (called number of intensity values (NoIV-1)), which can efficiently get rid of outliers. Furthermore, our work is plug-and-play in nature, so that it could be applied to diverse sample-based background subtraction methods. In experiments, we integrate our scheme into several state-of-the-art methods, and the results show that the performance of these methods in three indicators, recall, precision, and F-measure, improved from 4.95% to 16.47%, from 5.39% to 26.54%, and from 12.46% to 20.46%, respectively.

## 1. Introduction

Background modeling is the basis and also the first step for many vision-based tasks, like foreground detection, objects of interest segmentation, high-level operations [1,2,3,4] (such as target recognition and tracking, pose and gesture analysis, action understanding), etc. In the past decades, a gamut of approaches, ranging from simple methods of applying differencing of adjacent frames to the complex methods of mixing or combining multi-cues, have been proposed. Obviously, not only the accuracy of the background model in detection or other applications has been improved greatly, but the model itself has evolved by assimilating new techniques for pursuing higher efficiency and robustness.

Statistical theory has proven appropriate for modeling uncertainties of natural scenes [5], and in fact, it yielded the best results in CDnet2014 [6]. Generally, most of those methods based on statistics obey a suggestion from Stauffer and Grimson [7] that a recent sequence of observations is more important for parameters’ estimate, which somewhat coincides with the Markov property [8]. These observations are used for obtaining optimal parameters of the model in the training stage, whether online or offline, and then, these parameters are applied for initialization to guide the model to proceed elegantly. Subsequently, many researchers have determined this number *N* heuristically or by choosing *N* at point $N_{i}$, where the performance reaches the beginning of the plateau, from a range $\left(N_{0},N_{m}\right)$ $\left(N_{0}\;\leq N_{i}\;\leq N_{m}\right)$, experimentally. For example, Elgammal et al. [9] simply took a sample of size 100 to train and represent the background in their KDE model. The number of samples adopted analytically by Barnich and Droogenbroeck’s ViBemodel [10] and the SuBSENSEmodel of St-Charles et al. [11] was 20 and 35, respectively. We found that in CDnet 2014, SuBSENSE performed better than KDE and ViBe, while there were fewer samples needed in SuBSENSE than KDE and a few more than ViBe.

Therefore, a naturally-asked question is how many samples are needed after all and which samples are the best. All these questions point to an essential problem, which is the quality of samples (QoS) used for training or learning. To the best of our knowledge, QoS has not yet been focused on, and there is no principled way to settle it. In this paper, we tackle the two problems of “how many” samples and “which” samples of QoS.

To obtain an appropriate number in a principled way (that is the “how many” problem), we apply the tool variance, which is an important digital characteristic of random variables in statistics, on pixel values accumulatively (that is, each time from the first frame to the current frame for each pixel point). Thus, a series of variances is obtained, and the phenomenon of tending to converge at some frame emerges. However, for different pixel points, these variance sequences have different variance values where they converge, which can be seen from their distributions clearly, and this means that we cannot use a unified criterion to judge when they converge. We propose a new idea of applying the variance upon a window while sliding it on a previous variance sequence, which can tune the different values of corresponding converging points to a small positive value (such as 5${}^{-2}$), and so gets its name, bi-variance. The proof and more details will be given in Section 3.1.

The “which” problem mainly concerns the effect of noises. We know that noises are always present in videos and, thus, disturb the modeling process of normal intensity values. Methods in the literature either explicitly model the noise by the assumption of some distribution (for example, Gaussian with mean zero) or implicitly regard it as part of normal intensity values. However, noises are always there. To get rid of the noise, we construct the pixel histogram by counting the number of intensity values (NoIV-1) observed for each pixel point, and then at some time instant, we subtract one from those with a number greater than zero. This method obeys an underlying fact that the number of noises is less than that of normal intensity values in each pixel point over a period of time.

Summarily, the main contributions in this paper are: (1) a novel and effective convergent method, called Bi-Variance (BV), is proposed and proven in statistical and mathematical ways; and (2) a simple denoising method is introduced by subtracting one from the number of intensity values (NoIV-1); (3) our scheme can be easily integrated into other approaches thanks to its advantages of being plug-and-play; and (4) extensive tests are carried out to verify our proposals, and it turns out that the performance indicators of recall, precision, and F-measure are improved to different extents for several contemporary state-of-the-art models.

In the following, the content is organized as follows: the related work will be introduced firstly in Section 2; then, in Section 3, we will demonstrate our methods in detail and describe the algorithm in pseudocode; Section 4 is devoted to verifying the effectiveness of our scheme by applying it to several contemporary state-of-the-art models; finally, we will conclude our work and discuss how to make it perfect in our future work in Section 5.

## 2. Related Work

Background modeling of a real scene is a basic work for vision tasks. From about half a century ago to now, there have been numerous methods proposed for modeling the background [5,9,10,11,12,13,14,15,16,17,18] (a more detailed introduction about this technique and its applications was given in recent surveys [19,20,21,22,23]). However, most of them focus on how to choose the basic model (for example, Gaussian mixture model [7], kernel- or filter-based model [9,24], sample-based model [25,26,27,28,29], codebook model [30], etc.), the granularity of processing (for example, pixel-wise [13], blob-wise [31], region based [32,33], or spatiotemporal [5]), the components in a framework [10,11] (for example, one or more initializations, error propagation, and updating, apart from the essential subtraction process), and so on. In this paper, we do not come up with a new theory or method for background modeling, but propose a plug-and-play method to promote the performance of those approaches based on statistics.

Generally, a recent sample of a pixel’s intensity values is needed, denoted as $p_{1},p_{2},\cdots ,p_{N}$, where *N* is the number of values. Then, the model will be constructed by using this sample, and the incident pixel $p_{t}\;\left(t>N\right)$ will be judged as to which side it belongs, background or foreground. The question is how to decide the value of *N*. Toyama et al. [34] set a sequence with at least 200 frames for training the background model. Elgammal et al. [9,35] decided to use 100 samples to represent their background. Sheikh and Shah [5] straightforwardly ignored the discussion about this parameter and set it to 200 in their implementation. Barnich and Droogenbroeck [10] studied the influence of *N* by testing it from 2–50 and then set it to 20 because the curve of *N*-performance became flat as *N* became greater than 20. After their seminal work, recently, researchers have started to pay some attention to the tradeoff between the performance and computational costs by choosing an appropriate value for *N*. For instance, Bloisi and Iocchi [36] initialized it with 30 and then reduced it to 25. By performing an analog comparison like what ViBe [10] does, models like PBAS [13], LOBSTER [37], and SuBSENSE [11] all set their training sequence to 35 experimentally. However, the uncertainties of pixels are different from each other, so it is somewhat unreasonable to use the same number of samples for all pixels, let alone for all scenes. We propose a simple, but effective method to cope with this problem by deeply digging out the power of variance (please refer to Section 3.1).

In order to be consistently robust against various challenges, like illumination change, camouflage, etc., models should resolve well the influence by noises. In the literature, we can conclude that the noise is always treated in one of two ways, either explicitly or implicitly. The explicit way [11,24,31,35,38,39,40,41,42,43] means that the noise is modeled by using some measures, for instance a Gaussian distribution assumption. Tavakkoli et al. [44] claimed that PFinder [31] models each pixel with a single 3D Gaussian and models the noise, then detects pixels whose probabilities are smaller than a threshold chosen heuristically. Soatto et al. [38] assumed that a Gaussian white noise is involved in their description of dynamic texture, which is a generative model for the dynamic background [45]. Jing Zhong and Stan Sclaroff [24], in their ARMA, assumed that the noise was distributed as N(0,Q) where *Q* is its variance. Furthermore, a band-pass filter was used previously to account for the background changes over time. Crivelli et al. [41] proposed a so-called mixed state statistical framework. In this framework, they thought that background intensity values were perturbed by the Gaussian noise. However, unlike the aforementioned ways, they used a local average of intensity differences to reduce the influence of the noise. For computational consideration, López-Rubio and López-Rubio [39] utilized a diagonal matrix to model the noise, which was introduced by the pixel’s quantization and compression. Nikos and Visvanathan [46] utilized the spatial information to wipe out the disturbance of the noise. Thereafter, Li et al. [47] superadded the temporal information (that is, the spatio-temporal features) to further improve the ability against noises. The implicit way [5,10,37,48] always views the noise as part of the sources in modeling the background and sometimes adds a post-processing step, for example, to fill the holes, remove the blinking pixels, etc. Sheikh and Shah [5] applied a threshold *k* in their classifier to balance the trade-off between the sensitivity to the change and the robustness to the noise. Barnich and Droogenbroeck [10] claimed that their model can automatically adapt to the noise because it was constructed from noisy pixel values. SeungJong Noh and Moongu Jeon [48] and St-Charles and Bilodeau [37] simply applied pre- and post-processing to smooth or reduce the noise, respectively. However, Hofmann et al. [13] worried about the side effect of that, whether pre or post, which will also smooth blobs and lead to less sharp boundaries. Apart from these two types, Elgammal et al. [9,35,40] denoted that image noises can be modeled by a zero mean normal distribution if the scene is completely static, so only local or small global displacements, except for random noises, were addressed in their method.

No matter which case it is, the noise is always there. In this paper, we try to eliminate the noise to provide high-quality samples to train the model, and thus, it can be robust from the beginning. Section 3.2 will demonstrate our idea of removing the noise away in detail.

In the next section, the methodology, the main part of our paper, will be elaborated.

## 3. Methodology

In this section, firstly, we will give the detailed explanations (including motivations, proofs, effects, and analyses) of our methods: bi-variance and NoIV-1, in Section 3.1 and Section 3.2, respectively. Then, according to the descriptions presented in the former two parts, in Section 3.3, the corresponding algorithm is demonstrated in pseudocode.

### 3.1. Bi-Variance

**Figure 1:**
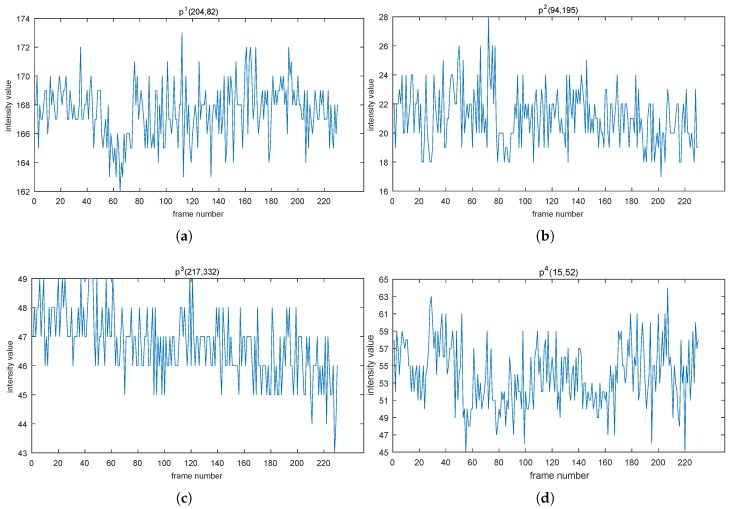
Points $p^{1}$(204, 82) in (**a**), $p^{2}$(94, 195) in (**b**), $p^{3}$(217, 332) in (**c**), and $p^{4}$(15, 52) in (**d**) are selected randomly from the scene bus station in category shadow [6]. Those four subfigures display the variation trend of the intensity values of the four points over a period of time. The *X*-axis’ name, frame number, indicates the order of each frame in a video in the temporal direction.

As we know, variance [49] is used to measure how far a set of (random) numbers is spread out from the average value. In the case of background modeling, we denote a recent set of intensity values of each pixel as $p_{1},p_{2},\cdots ,p_{n},\cdots ,p_{N}$, which are viewed as random numbers. Most of pixels’ intensity values behave with some underlying laws (see Figure 1), which motivates us to try to use the variance to model those values.

**Figure 2:**
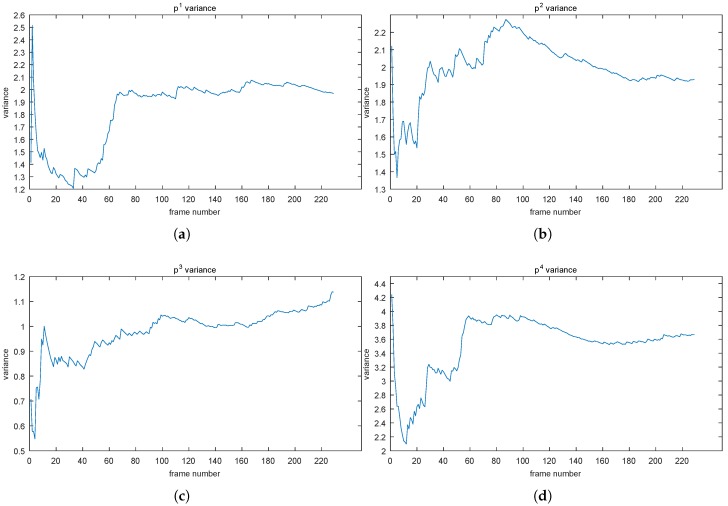
VCS tendencies of points mentioned in Figure 1 are showed in (**a**–**d**), separately. When the tendency reaches a stable status, the *Y*-axis value is also called the convergent value (cv).

It can be seen evidently that values of the pixel intensity fluctuate around some horizontal line and become stable asymptotically. to get the underlying law, we try to calculate variances of those cumulative sequences (like “$p_{1}$”, “$p_{1},\;p_{2}$”, “$p_{1},\;p_{2},\;p_{3}$”, etc.), which is defined as follows,

(1) $$VCS_{n}=\mathrm{var}\left[\left(p_{n}\right)\right],n\in \left[1,N\right]$$

where (·) represents a sequence [50] and var indicates the math operator “variance”. Thus, we can intuitively view the tendency of intensity values changing over time (see Figure 2).

As we expect, although they fluctuate drastically in the early stage, plateaus are always formed at some time instant. For instance, point $p^{1}$ became stable approximately at Frame 65, and the $VCS_{65}$ was two; point $p^{3}$ almost became stable at 10 with the $VCS_{10}$ being 0.9. So far, we can conclude, experimentally, that the VCS of each pixel intensity values was almost convergent and also call values like $VCS_{65}$ convergent values (cv). However, the cvs (2 for point $p^{1}$, 1.9 for point $p^{2}$, 0.9 for point $p^{3}$, and 3.6 for point $p^{4}$) were different, leading to a difficulty in judging when the convergent status (that is, the stable status) of each pixel intensity value was reached, which is related to resolving the “how many” sub-problem (the first problem of QoS). Therefore, we need a unified criterion to determine whether the sequence has achieved a convergent status.

Here, we propose a method, coined bi-variance, which views the VCS as a random variable, and thus, its value is the sequence $\left(VCS_{n}\right)$. Then, we set a window (with a size *L*, as 30 in our work) above the $\left(VCS_{n}\right)$ and also calculate the variance in each window by sliding along the $\left(VCS_{n}\right)$. In this way, we can obtain a unified criterion, zero, in theory (nevertheless, generally a small non-negative real value, like 5e-2 in our experiment). In the following, we will give proofs from two aspects, statistics and mathematics.

In statistics, assume that *X* is a random variable; thus, its expectation and variance are E(X) and D(X), respectively, and D(X) can be calculated as follows,

(2) $$D\left(X\right)=E\left(X^{2}\right)-{\left[E\left(X\right)\right]}^{2}.$$

Then, we define the bi-variance of *X* as B(X), and:

(3) B(X)=D(D(X)).

By replacing (2) into (3) and expanding it,

(4) $$\begin{array}{c} B\left(X\right)=E\left[D{\left(X\right)}^{2}\right]-E{\left[D\left(X\right)\right]}^{2}\\ =E\left\{{\left[E\left(X^{2}\right)-E{\left(X\right)}^{2}\right]}^{2}\right\}-{\left\{E\left[E\left(X^{2}\right)-E{\left(X\right)}^{2}\right]\right\}}^{2}\\ =E{\left(X^{2}\right)}^{2}-2E\left(X^{2}\right)E{\left(X\right)}^{2}+E{\left(X\right)}^{4}-E{\left(X^{2}\right)}^{2}\\ \;\;\;\;+2E\left(X^{2}\right)E{\left(X\right)}^{2}-E{\left(X\right)}^{4}\\ =0, \end{array}$$

we can see that no matter what the variance of *X* is, its bi-variance will tend to be zero.

From another perspective, according to (4),

(5) $$\begin{array}{c} B\left(X\right)=E\left[D{\left(X\right)}^{2}\right]-E{\left[D\left(X\right)\right]}^{2}\\ =\frac{1}{n}\sum_{i=1}^{n}D{\left(X_{i}\right)}^{2}-{\bar{D(X)}}^{2}\\ =\frac{1}{n}\sum_{i=1}^{n}{\left[D\left(X_{i}\right)-\bar{D(X)}\right]}^{2} \end{array}$$

where $\bar{D(X)}$ is the mean of sequence $\left(D\left(X_{n}\right)\right)$. From the last row of (5), $D\left(X_{i}\right)-\bar{D(X)}$ reflects the rate of $D(X_{i})$ deviating from the mean $\bar{D(X)}$, and from Figure 2, we know that the tendency of $D(X_{i})$ goes toward being stable, which means the deviation becomes increasingly weaker, and so does $D\left(X_{i}\right)-\bar{D(X)}$ (i.e., $D\left(X_{i}\right)-\bar{D(X)}$ approaching zero).

In mathematics, a series $S_{n}$ [51] can be defined by a sequence $\left(a_{n}\right)$ in its summation form,

(6) $$S_{n}=a_{1}+a_{2}+a_{3}+\cdots =\sum_{i=1}^{n}a_{i}$$

and a straightforward way [52] for judging whether $S_{n}$ is convergent is if the term $a_{i}$ reduces to zero with the *i* growing, meaning that from some position, like $i^{\prime }$, when $i>i^{\prime }$, $a_{i}\to 0$, and thus, the sum of this sequence, $S_{n}$, will tend to be a fixed value (that is, $S_{n}=a_{1}+a_{2}+a_{3}+\cdots +a_{i^{\prime }}$). Therefore, in (5), ${\left[D\left(X_{i}\right)-\bar{D(X)}\right]}^{2}$ can be viewed as a term of a sequence $\left({\left[D\left(X_{i}\right)-\bar{D(X)}\right]}^{2}\right)$, and thus, this sequence converges to zero (since $\lim_{n\to \infty }\frac{S_{n}}{n}=0$ with $S_{n}$ fixed).

**Figure 3:**
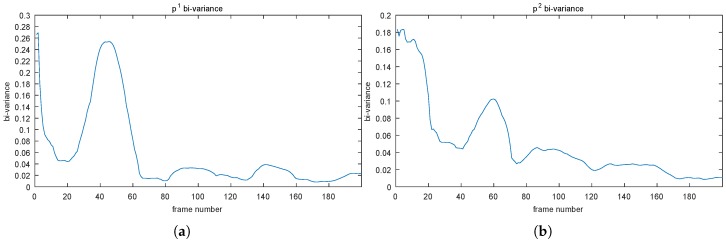

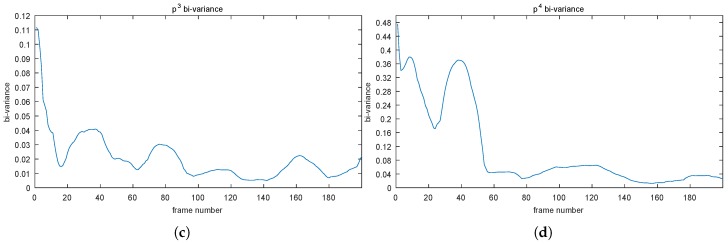
The bi-variance method is applied to the VCS of the four points displayed in Figure 2. Points $p^{1}$(204, 82) in (**a**), $p^{2}$(94, 195) in (**b**), $p^{3}$(217, 332) in (**c**), and $p^{4}$(15, 52) in (**d**).

By using the bi-variance on VCS in a window, we can get a new series of values. We call those values as “bi-variance”, which can converge in a unified way. Figure 3 illustrates the bi-variances of those VCS in Figure 2.

As mentioned before, we utilized a small positive value, 5${}^{-2}$, as the convergent criterion. Thus, those four points will converge approximately at Frames 65, 75, 10, and 55, respectively.

### 3.2. NoIV-1

**Figure 4:**
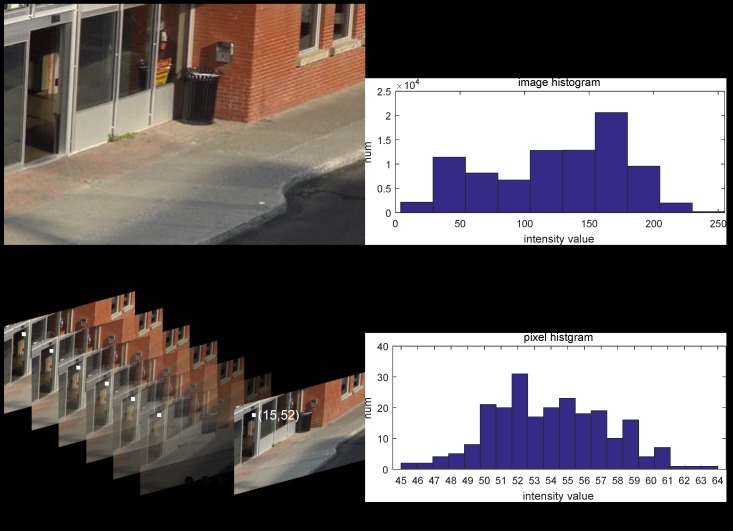
(**a**,**c**) display one image and an image sequence of the training set of the scene *bus station* in category **shadow**; (**b**,**d**) exhibit their histograms: image histogram and pixel histogram. Not like the image histogram, the range of pixel histogram always concentrates on a small interval, for instance from 45–64 in pixel (15, 52).

We know that the way of constructing the histogram of an image (e.g. Figure 4b) is to count the number of each intensity value (from 0–255) by scanning the whole image (Figure 4a). Different from the classic image histogram, a pixel histogram is established by counting the number of observed intensity values in the temporal axis (Figure 4c,d).

Further, by careful examination of Figure 4d, we can notice that the number of some intensity values (like 46, 63, 64) was very small, which is very important information from which our method derives.

**Figure 5:**
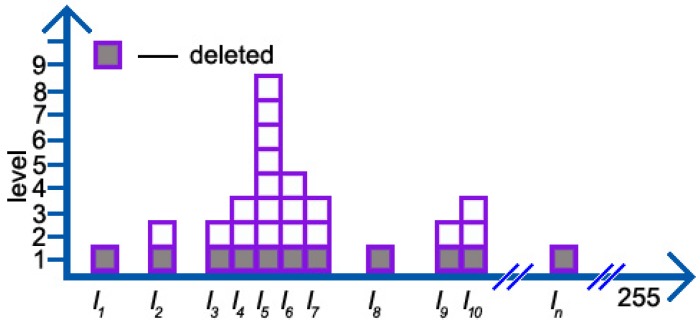
The number of squares in each column represents the number of intensity values in this column; for instance, the number of intensity values $I_{5}$ is eight. Squares in Level 1, colored in gray, will be deleted according to number of intensity values (NoIV-1).

Although we have no idea about when and where the noise will appear, we know that the number of normal intensity values is more than that of noises (normally, noises scatter in different places with the number of them being few during a short period). We simply cut each column by one in the pixel histogram (see Figure 5), leading to the disappearance of noises.

However, the effect of normal intensity values (for the case in Figure 5, $I_{1}$, $I_{8}$, and $I_{n}$ are viewed as noises, and others are normal) depends on their distribution. Therefore, analyses of it go as follows.

Assume that intensity values of one certain pixel *p* during a time period are $I_{1},\;I_{2},\;\cdots ,\;I_{n}$. The number of each intensity value is denoted by $N_{I_{1}},\;N_{I_{2}},\;\cdots ,\;N_{I_{n}}$. Thus, the total number of intensity values, $N_{T}$, is:

(7) $$N_{T}=N_{I_{1}}+N_{I_{2}}+\cdots +N_{I_{n}}.$$

Then, the contributions of each intensity value are:

(8) $$P_{I_{1}}=\frac{N_{I_{1}}}{N_{T}},\;P_{I_{2}}=\frac{N_{I_{2}}}{N_{T}},\;\cdots ,\;P_{I_{n}}=\frac{N_{I_{n}}}{N_{T}}.$$

After using NoIV-1, the number of intensity values is:

(9) $$N_{T}^{\prime }=\left(N_{I_{1}}-1\right)+\left(N_{I_{2}}-1\right)+\cdots +\left(N_{I_{n}}-1\right)=N_{T}-N_{L_{1}}$$

where $N_{L_{1}}$ indicates the number of the first-level pixel $p_{L_{1}}$ in the pixel histogram (for example, in Figure 5, the number of squares colored in gray in the bottom). According to (9), we will rewrite (8) as:

(10) $$P_{I_{1}}^{\prime }=\frac{N_{I_{1}}-1}{N_{T}^{\prime }},\;P_{I_{2}}^{\prime }=\frac{N_{I_{2}}-1}{N_{T}^{\prime }},\;\cdots ,\;P_{I_{n}}^{\prime }=\frac{N_{I_{n}}-1}{N_{T}^{\prime }}.$$

On the one hand, for those intensity values with the number being only one, their contributions will be reduced to zero, like $I_{1}$, $I_{8}$, and $I_{n}$ in Figure 5; on the other hand, for the others, we analyze the ratio by comparing their contributions presented in (8) and (10) as follows,

(11) $$ratio=\frac{P_{I_{i}}^{\prime }}{P_{I_{i}}}=\frac{\frac{N_{I_{i}}-1}{N_{T}^{\prime }}}{\frac{N_{I_{i}}}{N_{T}}}=\frac{N_{T}}{N_{T}-N_{L_{1}}}\cdot \frac{N_{I_{i}}-1}{N_{I_{i}}}$$

where $i\in p-p_{L_{1}}$. Let us have a look at the first item, $\frac{N_{T}}{N_{T}-N_{L_{1}}}$, in (11), in which the numerator is a constant and the denominator, in fact, can reflect the shape of the distribution. If $N_{L_{1}}$ is large, especially exceeding half of $N_{T}$, the shape always tends to be flat, otherwise, tall and narrow. For the other item, $\frac{N_{I_{i}}-1}{N_{I_{i}}}$, if $N_{I_{i}}=1$ (that is the noise), the effect of NoIV-1 is a fatal blow, or greater than one, $\lim_{N_{I_{i}}\to \infty }\frac{N_{I_{i}}-1}{N_{I_{i}}}\to 1$, meaning that the harm of those intensity values with a large number becomes weaker. Therefore, the contributions of the remaining intensity values mainly lie on the shape of their whole distribution, which is very important in determining an incident pixel belonging to the foreground or background in background models. As for the example in Figure 5, by applying NoIV-1, the contributions of noises, $I_{1}$, $I_{8}$, and $I_{n}$, become zero. Apart from those with only one remaining weakened intensity(that is $P_{I_{2}}^{\prime }$, $P_{I_{3}}^{\prime }$, and $P_{I_{9}}^{\prime }$ equal to $\frac{15}{19}$), the contribution of $P_{I_{4}}^{\prime }$, $P_{I_{7}}^{\prime }$, and $P_{I_{10}}^{\prime }$ is enhanced a little to be $\frac{60}{57}$, while $P_{I_{5}}^{\prime }$ and $P_{I_{6}}^{\prime }$ increase to $\frac{105}{76}$ and $\frac{45}{38}$, respectively.

**Figure 6:**
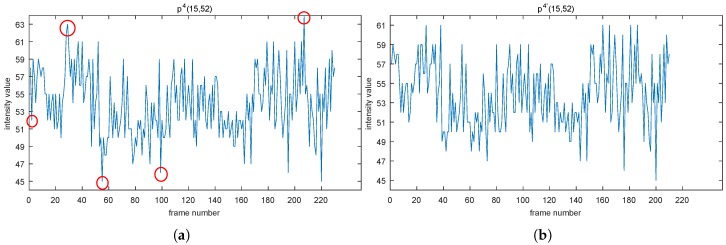
Distributions of $p^{4}$ presented in (**a**,**b**) are before and after using NoIV-1, respectively. Red circles in (a) remind about the disappearance of noises compared with (b).

In the real scene, by taking point $p^{4}$ for example, we can see that some peaks in Figure 6a (denoted by red circle) have disappeared compared to Figure 6b. Note that the point $p^{4}$ owns a unimodal distribution, which features a bump with contributions in two ends fewer than that of in the middle. That is why the points that have disappeared are located at the top or bottom in its distribution.

**Figure 7:**
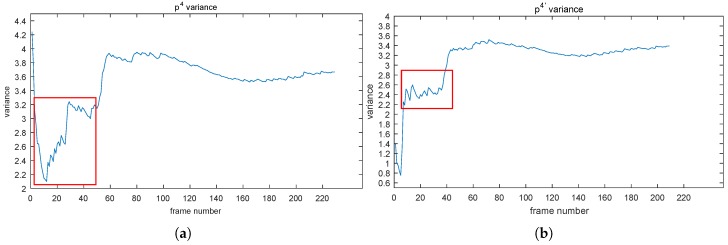

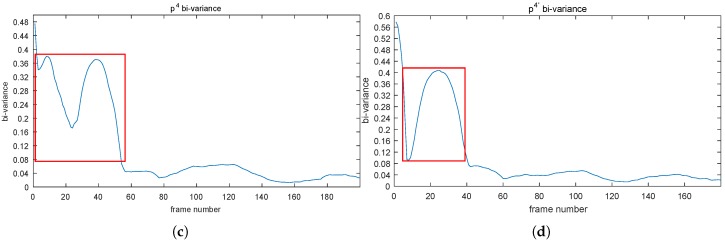
(**a**,**b**) depict the VCS curves of $p^{4}$ before and after using NoIV-1, respectively. (**c**,**d**) are the bi-variances of $p^{4}$ before and after using NoIV-1, respectively. Red rectangles emphasize the obvious changes.

The effect of NoIV-1 on the VCS of the point $p^{4}$ can be seen in Figure 7. In Figure 7a, we can see that the VCS curve of point $p^{4}$ had big changes from about 4.2, dropping down to 2.1 sharply, then rising to 4 with fluctuation and tended to be stable at about Frame 100. By using NoIV-1, the VCS curve (denoted by $p^{4^{\prime }}$ in Figure 7b) experienced fewer big changes and went into the convergent status at about Frame 50 quickly. The same effect was reflected by the bi-variance (see c and d, respectively).

However, an extreme case is that if the scene is very complex, like trees in a storm, the shape of its histogram will be very flat, and there would be may intensities in the bottom of the histogram. That means that all the intensities of the quantity being one in the bottom of the histogram will be viewed as noises and thus will be deleted according to the rule of NoIV-1. In this situation, one effective way is to maintain the reference samples long term.

### 3.3. Algorithm

According to the descriptions of our methods above, we will give the corresponding algorithm in pseudocode in Algorithm 1. Since the case of using bi-variance partnering with NoIV-1 performs better than that of using bi-variance alone (see Section 4), in our implementation, we placed NoIV-1 as the first step, then applied bi-variance on those purified results. Note that as we have not optimized our code, especially in NoIV-1, the whole runtime in the training stage of each host method was delayed from several milliseconds to tens of milliseconds (if the scene was challenging, leading to the sequence VCS converging slowly).

**Algorithm 1** The implementation of bi-variance and NoIV-1.
**Input:** an intensity value sequence $\left(p_{n}\right)$ of a pixel *P*, which is gathered from a scene video or image sequence during a period of time (for example, a set of frames, from the first image of any scene, with the amount specified in each scene’s configuration file in our experiment)**Output:** the new sequence $\left(p_{n}^{\prime }\right)$ without noises and the convergent frame cf1:Establish the pixelHistogram from $\left(p_{n}\right)$2:Remove the bottom level of the pixelHistogram3:Reconstruct the new sequence $\left(p_{n}^{\prime }\right)$4:
vcs_std=[] //to save the *VCS*
5:
$seqLen=(p_{n}^{\prime }).length$
6:
**for** i=1:seqLen **do**
7: $subSequence=\left(p_{n}^{\prime }\right)\left[1..i\right]$
8: vcs_std[i]=std[subSequence]
9:
**end for**
10:
window=L //the length of the sliding window
11:
cnt=0 //to record consecutive times of the bi-variance below ϵ, which is introduced in the following
12:
cnt_thres=THRESHOLD //the threshold of cnt
13:
cf=seqLen //initialized for the non-convergent case
14:
wstd=0 //the variance of vcs_std in the window, i.e. the bi-variance
15:
**for** j=window:seqLen **do**
16: wstd=std(vcs_std(j−window+1:j)) //ϵ, a small positive real value, like 5e-217: **if** wstd<ϵ **then**
18:  cnt=cnt+119:  **if** cnt<cnt_thres **then**
20:   cf=j−cnt_thres21:   break22:  **else**23:   cnt=024:  **end if**25: **end if**26:
**end for**
27:**return**$\left(p_{n}^{\prime }\right)$, cf


## 4. Experiment

To validate the effectiveness of our methods, We tested our method on a PC with a 3.3-GHz Intel Xeon E3-1230 V2 380 CPU and 24 GB memory, and the environment was Microsoft Visual Studio 2010. We performed extensive tests on a well-known dataset (CDnet 2014) [6], which is adopted widely by researchers in studying background modeling and other works related to computer vision. CDnet 2014 involves eleven categories and 4–6 scenes in each one (53 scenes in total). There were three performance indicators, recall, precision, and F-measure, which are often used in the literature, and so, we also used them in this work. Their definitions are:

(12) $$\begin{array}{c} Recall=\frac{TP}{TP+FN}\\ Precision=\frac{TP}{TP+FP}\\ F-Measure=2\cdot \frac{Recall\cdot Precision}{Recall+Precision}=\frac{2TP}{2TP+FN+FP} \end{array}$$

where TP, FP, TN, and FN indicate the number of “true positives”, “false positives”, “true negatives”, and “false negatives”, respectively.

Four famous methods (KDE [9], ViBe [10], PBAS [13], SuBSENSE [11]) in the recent two decades were chosen to take the role of a carrier (as claimed before, our methods can be viewed as a plug-in).

Then, we set three scenarios for evaluating the performance of our methods:

1. The basic one is that the original codes of these methods were executed without any modification. Note that because the starting points of the test of each method were different (such as KDE and SuBSENSE testing after their training frames, while ViBe from the first frame and PBAS with the first one blank), we conventionally obeyed the rule of setting the training number, which is in each scene’s configuration file of CDnet 2014, as the starting points of the test.
2. The second test utilized the bi-variance to get the “right” number of frames for training based on the basic one.
3. The third scenario was a combination of the basic one with our methods, bi-variance and NoIV-1.

### 4.1. Quantitative Results and Analysis

The quantitative results of four host methods are listed in Table 1, Table 2, Table 3 and Table 4, respectively. The last row with the title *ratio* indicates the performance enhancement by comparing the best results of each method after using *bi-variance and NoIV-1* against the basic one.

**Figure 8:**
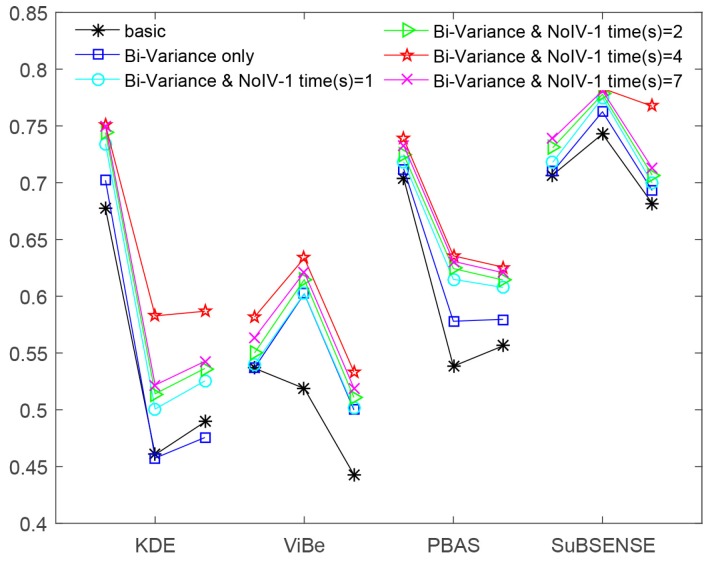
Results in Table 1, Table 2, Table 3 and Table 4 are drawn here. In each broken line, the three vertices stand for *recall*, *precision*, and *F-measure*, from left to right.

Meanwhile, to provide intuitive knowledge, we depict all of these data in Figure 8.

In Table 1, Table 2, Table 3 and Table 4, column “times” was only used for the third scenario, in which the method NoIV-1 was executed one time, two times, four times, and seven times, respectively. Firstly, we can notice that the performance of all host methods in the three indicators was enhanced by using *bi-variance only* or that together with NoIV-1. Secondly, the effects of applying their combination, *bi-variance and NoIV-1*, were better than those of only utilizing bi-variance. Thirdly, we found that when the method NoIV-1 was executed a different number of times, the final results were different. However, when we executed it four times, each host method in the combined model reached its best performance, and if we executed it three more times, the performance went down.

**Figure 9:**
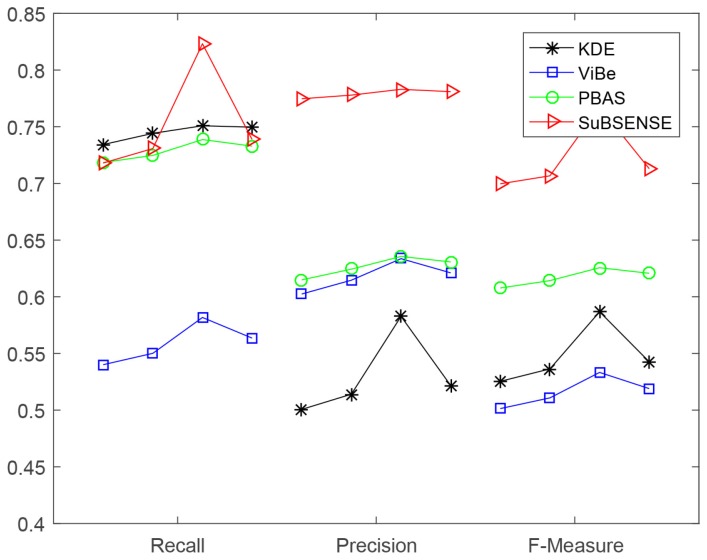
The tendency of each performance indicator of each host method by executing method “NoIV-1” one time, two times, four times, and seven times, respectively, in the combined model. In each broken line, the four vertices represent the four kinds of the different number of times of executing the method “NoIV-1”.

These phenomena reveal two facts: (1) the noise is a key factor in affecting the precision of background models; (2) the number of noises is a small number. By viewing Figure 9 below, there is a tendency of rising first and then descending for each method’s performance indicator. Recall Figure 5 and the analyses in Section 3.2: executing method “NoIV-1” more times was increasingly harmful to the normal intensities and would destroy (rather than restore) the distribution of data. Therefore, the tendencies in Figure 9 are reasonable, and the tendency was basically predictable if we executed the method “NoIV-1” more than seven times.

In Table 1, for method *KDE*, the values of the indicators *precision* and *F-measure* of *bi-variance only* are smaller than that of *basic* (visually in Figure 8, part of *bi-variance only*’s line below that of *basic*), because the training number of frames of *bi-variance only* was more than that of *basic* (100 mentioned in Section 2), leading to more noises being introduced.

In Table 2 and Table 3, the *ratio*s of performance enhancement in *recall* are less than those in *precision* and *F-measure*, which shows that our methods effectively reduced the number of FP more than that of FN by referring to their definitions in (12).

Interestingly, in Figure 8, we can also find that SuBSENSE had the best performance in all three scenarios against the others, which is a more recent method and gained a very high ranking in CDnet 2014 [6]. ViBe’s performance obtained a relatively big promotion when our methods were applied to its training stage, which actually reflected that the influence of noises cannot be ignored and that an explicit processing will be better (for example, SuBSENSE adopts an explicit way, while ViBe’s denoising mechanism is by constructing the model from noisy data straightforwardly).

### 4.2. Qualitative Results and Analysis

**Figure 10:**
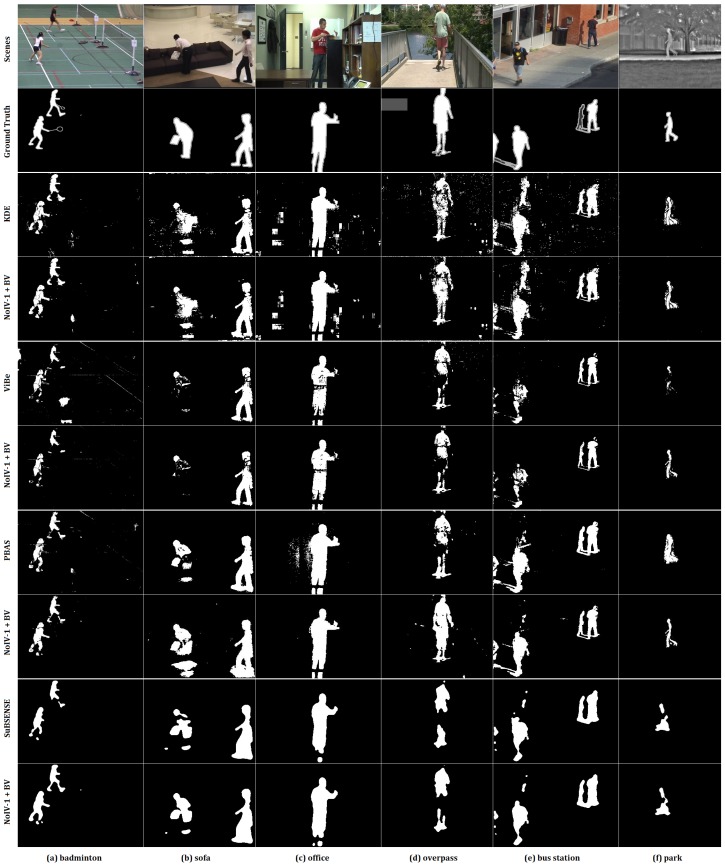
Scenes from column (**a**–**f**) are from categories **camerJitter**, **intermittentObjectMotion**, **baseline**, **dynamicBackground**, **shadow**, and **thermal**, respectively.

The qualitative results (see Figure 10 below) will display each method’s cons and pros visually, especially in detail.

In Figure 10, for the results of the method KDE in the third row compared with those of KDE combined with our methods in the forth row, the obvious change is that the noise was depressed greatly (especially in the last three scenes).

For methods ViBe and PBAS, the promotion was relatively higher than that in the other two host methods, which can also be noticed in their quantitative results in Figure 8. For example, the method ViBe cannot reduce the disturbance of white lines in the badminton court of the scene badminton and the white pattern on the T-shirt of the scene office. However, those problems were alleviated after using our methods. Method PBAS was susceptible to illumination change, the dynamic background, and the thermal environment. Those can be identified distinctly in scenes office, overpass, and park in the seventh and eighth row.

As for the method SuBSENSE, one of its drawback is overfitting, which can be viewed in the last two rows; for instance, the one missing leg in scenes badminton and office, the separated body in scenes sofa and park, and the connected legs in the scene overpass.

### 4.3. Convergent Frames and Analysis

Table 5 gives the convergent frames (that is, the desirable number of training frames) of all scenes in each category in CDnet 2014 [6].

By comparing those convergent frames of different scenes, there are several highlights presented in Table 5:

1. the convergent frame of every scene was totally different from the others;
2. there was a big gap between the minimal convergent frame, 32, and the maximal convergent frame, 380, of the whole scenes;
3. the challenging scenes, like badWeather, dynamicBackground, PTZ, turbulence, etc., evolved longer to obtain convergence.

Therefore, we assert that the arrangement of assigning all scenes with the same *N* is not appropriate, and the exact value of *N* should be determined by the data themselves. Additionally, The runtime of our method was from about 20 ms to less than 2 s, which specifically depended on the complexity, the resolution, and the convergent frame of each scene. Since we directly plugged our method into the source code of each host method, it exactly led to a delay to some extent. We are also considering this issue and intend to optimize our approach for speedup.

## 5. Conclusions

In this paper, we focus on the quality of training samples (QoS) for background modeling, and we have proposed a plug-and-play method, bi-variance, to cope with the “how many” problem in QoS and coupled with another denoising method, called NoIV-1, to sweep away those outliers, which refers to the “which” problem of QoS. Four seminal works, KDE [9], ViBe [10], PBAS [13], and SuBSENSE [11], have been introduced to evaluate our methods. In the experiment, we can see that the performance of each method (in three indicators, recall, precision, and F-measure) has been enhanced to different degrees.

By viewing the ratio-rows from Table 1, Table 2, Table 3 and Table 4, the ratio of performance promotion was limited, generally speaking. However, how to greatly improve the performance is concerned with the quality of background model itself. Consequently, in our future work, we need to propose a high-quality background model based on the current study.

## Figures and Tables

**Table 1:** Results of KDE executed on three scenarios.

Scenario	Times	Recall	Precision	F-Measure
basic	-	0.677320	0.460483	0.489317
Bi-Variance only	-	0.702139	0.457460	0.475546
Bi-Variance and NoIV-1	1	0.734267	0.500651	0.525397
	2	0.744162	0.514061	0.536388
	4	**0.750847**	**0.582704**	**0.586779**
	7	0.749623	0.521745	0.542624
ratio	-	10.86%	26.54%	19.92%

**Table 2:** Results of ViBeexecuted on three scenarios.

Scenario	Times	Recall	Precision	F-Measure
basic	-	0.536554	0.518769	0.442704
Bi-Variance only	-	0.536887	0.602184	0.499898
Bi-Variance and NoIV-1	1	0.540093	0.602429	0.501373
	2	0.550113	0.614631	0.510812
	4	**0.581746**	**0.633618**	**0.533265**
	7	0.563545	0.621112	0.518979
ratio	-	8.42%	22.14%	20.46%

**Table 3:** Results of PBASexecuted on three scenarios.

Scenario	Times	Recall	Precision	F-Measure
basic	-	0.703802	0.538357	0.556289
Bi-Variance only	-	0.712157	0.577949	0.579673
Bi-Variance and NoIV-1	1	0.718419	0.614902	0.607733
	2	0.724719	0.624375	0.614255
	4	**0.738673**	**0.635568**	**0.625628**
	7	0.732899	0.630746	0.620714
ratio	-	4.95%	18.01%	12.46%

**Table 4:** Results of SuBSENSEexecuted on three scenarios.

Scenario	Times	Recall	Precision	F-Measure
basic	-	0.706819	0.742927	0.681875
Bi-Variance only	-	0.709695	0.762506	0.693169
Bi-Variance and NoIV-1	1	0.717996	0.774682	0.699669
	2	0.730749	0.777897	0.706701
	4	**0.823209**	**0.783015**	**0.767367**
	7	0.738729	0.780936	0.712979
ratio	-	16.47%	5.39%	12.54%

**Table 5:** Convergent frames of all scenes in each category in CDnet 2014.

Category	Scene	Convergent Frame
**badWeather**	blizzard skating snowFall wetSnow	38 125 66 139
**baseline**	highway office pedestrians PETS2006	64 60 58 51
**cameraJitter**	badminton boulevard sidewalk traffic	116 92 88 96
**dynamic Background**	boats canoe fall fountain01 fountain02 overpass	237 32 87 67 124 141
**intermittent ObjectMotion**	abandonedBox parking sofa streetLight tramstop winterDriveway	154 76 62 38 172 132
**lowFramerate**	port_0_17fps tramCrossroad_1fps tunnelExit_0_35fps turnpike_0_5fps	233 89 238 118
**nightVideos**	bridgeEnry busyBoulevard fluidHighway streetCornerAtNight tramStation winterStreet	218 163 43 152 116 214
**PTZ**	continuousPan intermittentPan twoPositionPTZCam zoomInZoomOut	60 195 79 59
**shadow**	backdoor bungalows busStation copyMachine cubicle peopleInShade	48 37 166 85 128 80
**thermal**	corridor diningRoom lakeSide library park	44 47 42 40 104
**turbulence**	turbulence0 turbulence1 turbulence2 turbulence3	373 380 149 338
